# The association of aerobic, resistance, and combined exercises with the handgrip strength of middle-aged and elderly Korean adults: a nationwide cross-sectional study

**DOI:** 10.1186/s12877-022-03293-z

**Published:** 2022-08-16

**Authors:** Joo Hye Sung, Se Rhim Son, Seol-Hee Baek, Byung-Jo Kim

**Affiliations:** 1 Department of Neurology, Korea University Anam Hospital, Korea University College of Medicine, 73, Goryeodae-ro, Seongbuk-gu, Seoul, 02841 Republic of Korea; 2 Department of Biostatistics, Korea University College of Medicine, Seoul, Republic of Korea; 3 BK21FOUR R&E Center for Learning Health Systems, Korea University, Seoul, Republic of Korea

**Keywords:** Physical activity, Handgrip strength, Sarcopenia, Korean national health, Cross-sectional study

## Abstract

**Background:**

Handgrip strength (HGS), an indicator of overall muscle strength, is a key component in sarcopenia diagnosis. Although exercise is an effective strategy to prevent sarcopenia, the most appropriate exercise type targeting sarcopenia needs to be established. This study aimed to investigate the relationship between the physical activity (PA) patterns and HGS.

**Methods:**

This was a cross-sectional study using the data from the 7th Korea National Health and Nutrition Examination Survey (2016–2018). The study population included 12,814 adults aged ≥ 40 years. According to the World Health Organization PA guidelines for public health, both aerobic (moderate to vigorous PA ≥ 150 min/week) and resistance exercises (≥ 2 sessions/week) are recommended. Study participants were categorized into one of the four groups depending on their adherence to each of two exercise guidelines (“neither,” “aerobic only,” “resistance only,” and “combined”). By defining normal HGS cutoff values as the lowest quartile of HGS from the population aged 20 years and above, we classified participants as “preserved” HGS group if their HGS was equal to or above the cutoff values. A Poisson regression model was used to calculate adjusted prevalence ratios (APRs) for preserved HGS across the four PA guideline adherences stratified by age and sex groups.

**Results:**

In middle-aged adults, the “combined” exercise group was independently associated with the preserved HGS (male, age 50–59 years, APR = 1.072; male, age 60–69 years, APR = 1.180; female, age 50–59 years, APR = 1.112; female, age 60–69 years, APR = 1.188). For adults aged ≥ 70 years, meeting only aerobic or resistance exercise guidelines showed a positive association with HGS before adjusting for other health-related variables. In males of ≥ 70 years, the APR of preserved HGS was highest in the “combined” exercise group (“resistance only,” APR = 1.459, “combined,” APR = 1.664), while in women aged ≥ 70 years, the significance was lost after adjusting for covariates.

**Conclusions:**

Adults meeting both aerobic and resistance exercise guidelines were associated with the highest prevalence of preserved HGS. Performing both types of exercise might be the most effective way to prevent sarcopenia that should be investigated in future clinical trials.

**Supplementary Information:**

The online version contains supplementary material available at 10.1186/s12877-022-03293-z.

## Introduction

Sarcopenia is a condition associated with accelerated age-related skeletal muscle loss, and leads to a higher risk of falls and fractures, difficulty in daily activities, and negative impact on quality of life [1]. The decline in muscle mass starts from the age of 40 years, while sarcopenia is highly prevalent in the older population [2, 3]. It is reported that approximately 3.2 to 26.3% of the population aged 60 years or above are suffering from sarcopenia [4]. With a rapidly aging population worldwide, sarcopenia is likely to create a significant burden on the public health system in the near future. According to the definition proposed by the European Working Group on Sarcopenia in Older People (EWGSOP2) [5], clinical suspicion of sarcopenia starts with muscle strength assessment, usually handgrip strength (HGS), which has been proven to be a more powerful predictor of adverse health outcomes than muscle mass [1, 6]. HGS is a screening tool for sarcopenia, commonly used in research and clinical settings to measure muscle strength due to its well-validated protocol and cost-effectiveness [7]. HGS is an indicator of overall health status in the older population, which was shown to be significantly associated with disability and all-cause mortality in elderly [8, 9].

The positive role of exercise training in treating and preventing sarcopenia has been widely accepted [10–12]. Research has been conducted targeting healthy elderly and sarcopenia patients, providing compelling evidence toward the beneficial effects of exercise on muscle strength, muscle mass, and physical performance [13–16]. Among the different types of exercise programs, resistance exercise is the mainstream intervention for maintaining skeletal muscle health [17–20]. Conversely, interest in aerobic exercise has been mainly focused on improving cardiorespiratory capacity. However, many studies have reported improved muscle growth [21, 22] and strength [23] after aerobic exercise. Furthermore, a combined exercise program composed of both resistance and aerobic exercise training has shown greater improvement in gaining muscle strength and improving physical performance compared to either mode alone [24–26].

Despite the accumulating evidence, the optimal strategy for designing a physical exercise program to prevent sarcopenia remains unclear [27]. Sex and age are also important factors influencing the effects of exercise intervention [28, 29]. Investigating the relationship between physical activity (PA) patterns and HGS in a large-scale population would be helpful in establishing a preventive strategy against sarcopenia. Therefore, our study aimed to investigate PA pattern-based differences in HGS using a nationally representative dataset from Korea. Since the muscle strength starts to decline after the age of 40 years, we selected subjects aged 40 years or above, and investigated the association of different PA patterns with HGS across different sex and age groups.

## Materials and methods

### Data collection and participants

We performed this study using data from the 7th Korea National Health and Nutrition Examination Survey (KNHANES, 2016–2018) conducted by trained specialists under the supervision of the Korea Centers for Disease Control and Prevention (KCDC). The KNHANES is a nationwide, multistage-stratified, and complex design survey on the health and nutrition of a representative sample of the entire South Korean population. It consists of a health questionnaire, examination, and nutrition survey. The health questionnaire and examination surveys were conducted at mobile examination centers, while the nutrition survey was conducted by visiting each household unit in person. In the health questionnaire surveys, data on sociodemographic variables, medical history, and the entire nutrition records were collected through face-to-face interviews. The entire nutrition records involve information about the type of food consumed over the past 24 h including how it was prepared, processed, and the weight of each ingredient. Data on health-related behaviors, including PA, smoking, and drinking, were collected using self-reported questionnaires.

Of the 24,269 participants in the 2016–2018 survey, 13,959 adults aged 40 years and above were initially selected. After excluding those with missing data for HGS (1,145 participants), 12,814 participants (5,646 men, 7,168 women) were finally included in our analysis. The numbers of missing values for each variable of interest other than HGS were as follows: PA (*n* = 653); education level (*n* = 629); income level (*n* = 48); alcohol consumption (*n* = 140); smoking status (*n* = 150); medical history of dyslipidemia (*n* = 8); body mass index (BMI) (*n* = 39); and nutritional status (*n* = 1618). Data collection was performed after approval by the Institutional Review Board of the KCDC (approval number: 2018–01-03-P-A) and conducted in accordance with the Declaration of Helsinki. The requirement for informed consent was waived by Korean Centers for Disease Control and Prevention ethics committee as anonymous and de-identified information was used. The full description of the survey and dataset used in this study is available in a public open-access KNHANES repository. (https://knhanes.kdca.go.kr/knhanes/sub03/sub03_02_05.do).

### Handgrip strength

A digital grip strength dynamometer (TKK 5401; Takei Scientific Instruments Co., Ltd., Tokyo, Japan) was used to measure HGS. Subjects with structural deformities on hand/wrist, or with a history of hand/wrist surgery within the last 3 months or hand/wrist pain within the last week were excluded from the measurement. HGS was measured while the participants were in a standing position with their arms hanging naturally at the height of their thigh without bending their elbow or wrist. Measurements were taken over a duration of up to 3 s. Three measurements were taken from each hand, and the maximum value among the six measurements was used as the final measurement of the HGS. Normal HGS cutoff values was defined on the basis of the lowest quartile of HGS from the total population (*n* = 17,610, 7,808 men, 9,802 women) aged 20 years and above from the original 7th KNHANES dataset (34.4 kg for men, 20.0 kg for women). We classified participants as “reduced” HGS group if their HGS was below the cutoff values, and as “preserved” HGS group if their HGS was equal or above the cutoff values.

We did not use the EWGSOP2 proposed cutoff values for reduced HGS (27 kg for men, 16 kg for women) as there was a relatively small number of subjects who belong to “reduced HGS” group when this criterion was applied (1210 out of 12,814 which represents only 9.4% of the total sample). Furthermore, in the 40–49 years age group, the number of subjects belonging to “reduced HGS” group became even smaller (male: 15 out of 1368, 1.1%; female: 37 out of 1774, 2.1%).

### Physical activity assessment

The participants’ degree of regular exercise was assessed using the Korean version of the modified Global Physical Activity Questionnaire (K–GPAQ) [30, 31]. The GPAQ, developed by the World Health Organization (WHO) for PA surveillance, was translated into a Korean version in 2013, with established reliability and validity [32]. The aerobic exercise level performed by the participants was estimated by calculating the amount of moderate-to-vigorous-intensity physical activity (MVPA). The vigorous-intensity PA refers to activities that require hard physical effort and cause large increases in breathing or heart rate, whereas the moderate intensity PA refers to activities that require moderate physical effort and cause small increases in breathing or heart rate. Respondents were asked to report the frequency (days) and duration (hours or minutes) of moderate-intensity and vigorous-intensity PA in a typical week and were asked to report only activities that lasted for at least 10 continuous minutes. The total MVPA level was calculated by multiplying the frequency and duration, while the minutes spent on vigorous-intensity PA were multiplied by two. To assess resistance exercise performance, respondents were asked, “Over the past 7 days, how many days did you do any physical activities specifically designed to strengthen your muscles such as sit-ups, push-ups, lifting weights or dumbbells?” [33].

The year 2010 WHO “Global recommendations on physical activity for health” recommends that adults over the age of 18 years should engage in (1) ≥ 150 min/week of moderate-intensity aerobic PA, or ≥ 75 min/week of vigorous-intensity aerobic PA, or an equivalent combination of both; and (2) resistance exercise at a moderate or greater intensity that involves all the major muscle groups for at least two or more days a week. The study participants were categorized into one of the four groups depending on their adherence to each of two exercise guidelines based on the aforementioned WHO guideline: “neither” (MVPA 0–149 min/week and resistance exercise 0–1 sessions/week); “aerobic only” (MVPA ≥ 150 min/week and resistance exercise 0–1 sessions/week); “resistance only” (MVPA 0–149 min/week and resistance exercise ≥ 2 sessions/week); and “combined” (MVPA ≥ 150 min/week and resistance exercise ≥ 2 sessions/week).

### Covariates

Data on sociodemographic variables and health-related factors that might be related to HGS were collected using standardized survey items. The sociodemographic variables included age, sex, and education level (“below middle school graduate,” “high school graduate,” or “college graduate or higher”), location of residence (“urban” or “rural”), living situation (“single-person household” or “with members”), and quartiles of household income. Health-related factors included smoking status (“never smoker,” “ex-smoker,” or “current smoker”), alcohol consumption (“ < 4 times/month” or “ ≥ twice/week”), and presence of any comorbidities (hypertension, diabetes mellitus, or dyslipidemia) from the medical history. Information regarding past medical history was acquired by asking the subjects if they had ever been diagnosed with a certain disease condition. BMI was calculated from the objectively measured height (m) and weight (kg). The nutrition survey was conducted using open-ended questionnaires to record total food consumed over the past 24 h using the 24 h recall method. The amounts of total energy and macronutrient (i.e. protein, fat, carbohydrates) intakes were calculated by referencing the nutrient contents of foods described in the Korean Food Composition Table.

### Statistical analysis

Categorical variables were expressed as frequency and percentage (%), whereas continuous variables were presented as mean and standard deviation. Sociodemographic and health-related factors were compared between the groups using the chi-square test for categorical variables and t-test for continuous variables. Analysis of variance with post-hoc analysis was used to compare continuous variables between more than two groups. Poisson regressions with a robust error variance were used to calculate prevalence ratios (PRs) for preserved HGS across the four PA guideline adherence categories (i.e. “neither,” “aerobic only,” “resistance only,” and “combined”). For these analyses, not meeting either “aerobic” and “resistance” exercise guidelines (“neither”) were used as the reference group. Sex- and age-stratified analyses were performed to observe differences between the sex and age groups. PRs were reported with models that were unadjusted and adjusted for health-related factors. A sensitivity analysis using the EWGSOP2 proposed HGS cutoff values are presented as Supplemental Table 1. Prior to conducting our final analytical models, we assessed collinearity among covariates using tests for the variance inflation factor (VIF), with a VIF ≥ 5 indicating multi-collinearity. Two pairs of covariates contained VIFs ≥ 5: “energy intake” and “fat intake,” “energy intake” and “carbohydrate intake.” After excluding “energy intake,” the VIFs ranged from 1.07 − 2.54, indicating no evidence of collinearity. The statistical analyses were performed using SAS version 9.4 (SAS Institute, Inc., Cary, NC, USA), and *p* values < 0.05 were considered statistically significant.

## Results

### Differences between “reduced” and “preserved” handgrip strength groups

To analyze general characteristics, subjects were divided into the “preserved” and “reduced” HGS groups for men and women separately. Most of the studied sociodemographic and health-related factors differed significantly between the groups (Table 1). The subjects in the “reduced HGS” group were significantly older (men, 68.0 ± 10.9 years vs 55.7 ± 10.1 years, *p* < 0.001; women, 67.3 ± 11.6 years vs 56.3 ± 10.5 years, *p* < 0.001). Compared to the “preserved HGS” group, the proportions of subjects with low education and income levels were higher in the “reduced HGS” group in both sexes (*p* < 0.001 for both the parameters). Furthermore, “reduced HGS” group contained more rural residents and single-person households than urban residents and with family members in both sexes (*p* < 0.001 for both the parameters). The male subjects in the “reduced HGS” group showed significantly lower BMI compared to those in the “preserved HGS” group (23.4 ± 3.1 kg/m^2^ vs 24.7 ± 2.9 kg/m^2^, *p* < 0.001). Male smokers and heavy drinkers from both sexes were more prevalent in the “preserved HGS” group than in the “reduced HGS” group (*p* < 0.001 for both the parameters). A significantly lower intake of total energy, protein, fat, and carbohydrate was observed in the “reduced HGS” group compared to the “preserved HGS” group in both sexes (all *p* < 0.001). Hypertension and diabetes mellitus from both sexes and dyslipidemia in females were more prevalent in the “reduced HGS” group than in the “preserved HGS” (all *p* < 0.001).

**Table 1:** Characteristics of the study population of the preserved and reduced HGS groups by sex

	**Male (** ***n*** ** = 5646)**			**Female (** ***n*** ** = 7168)**		
	**Reduced HGS (** ***n*** ** = 1688)**	**Preserved HGS (** ***n*** ** = 3958)**	***p*** ** value**	**Reduced HGS (** ***n*** ** = 2032)**	**Preserved HGS (** ***n*** ** = 5136)**	***p*** ** value**
Age, years (mean ± SD)	68.0 ± 10.9	55.7 ± 10.1	< 0.001	67.3 ± 11.6	56.3 ± 10.5	< 0.001
Education level, n (%)			< 0.001			< 0.001
Below middle school graduate	791 (50.3)	816 (21.8)		1302 (68.2)	1804 (36.4)	
High school graduate	384 (24.4)	1307 (34.9)		337 (17.7)	1707 (34.4)	
College graduate or higher	399 (25.4)	1619 (43.3)		269 (14.1)	1450 (29.2)	
Location of residence, n (%)			< 0.001			< 0.001
Urban	1251 (74.1)	3172 (80.1)		1471 (72.4)	4228 (82.3)	
Rural	437 (25.9)	786 (19.9)		561 (27.6)	908 (17.7)	
Living situation, n (%)			< 0.001			< 0.001
Single	246 (14.6)	364 (9.2)		522 (25.7)	627 (12.2)	
With family members	1442 (85.4)	3594 (90.8)		1510 (74.3)	4509 (87.8)	
Quartiles of income, n (%)			< 0.001			< 0.001
1^st^ (lowest)	503 (30.1)	864 (21.9)		569 (28.2)	1199 (23.4)	
2^nd^	410 (24.5)	996 (25.2)		524 (26.0)	1269 (24.8)	
3^rd^	380 (22.7)	1045 (26.5)		447 (22.1)	1321 (25.8)	
4^th^ (highest)	379 (22.7)	1044 (26.4)		479 (23.7)	1337 (26.1)	
BMI, kg/m^2^ (mean ± SD)	23.4 ± 3.1	24.7 ± 2.9	< 0.001	24.0 ± 3.6	24.0 ± 3.5	0.706
Smoking status, n (%)			< 0.001			0.154
Never smoker	324 (19.5)	698 (17.8)		1842 (92.7)	4657 (91.3)	
Ex-smoker	887 (53.5)	1867 (47.7)		73 (3.7)	220 (4.3)	
Current smoker	448 (27.0)	1351 (34.5)		72 (3.6)	224 (4.4)	
Alcohol consumption (frequency), n (%)			< 0.001			0.008
< 4/month	991 (64.6)	2186 (58.3)		1206 (90.3)	3734 (87.7)	
≥ 2/week	544 (35.4)	1564 (41.7)		129 (9.7)	526 (12.4)	
Nutritional status						
Energy intake (Kcal)	1899.8 ± 756.7	2309.0 ± 905.2	< 0.001	1432.0 ± 600.4	1641.5 ± 656.1	< 0.001
Protein intake (g)	64.5 ± 33.3	82.7 ± 41.5	< 0.001	47.9 ± 26.0	58.9 ± 29.2	< 0.001
Fat intake (g)	33.1 ± 26.6	48.6 ± 34.4	< 0.001	25.2 ± 21.5	35.2 ± 25.8	< 0.001
Carbohydrate intake (g)	311.2 ± 117.1	344.2 ± 128.4	< 0.001	255.4 ± 105.8	272.0 ± 114.3	< 0.001
Comorbidities						
Hypertension, n (%)	778 (46.1)	1212 (30.6)	< 0.001	916 (45.2)	1319 (25.7)	< 0.001
Dyslipidemia, n (%)	352 (20.9)	837 (21.2)	0.825	643 (31.7)	1280 (24.9)	< 0.001
Diabetes mellitus, n (%)	368 (21.8)	464 (11.7)	< 0.001	379 (18.7)	453 (8.8)	< 0.001

Continuous variables were expressed as mean ± standard deviation, and categorical variables were expressed as numbers (percentages). *p* values were derived using the chi-square test or t-test. Reduced HGS was defined as HGS < 34.4 kg in men and HGS < 20.0 kg in women

*HGS* handgrip strength, *SD* standard deviation

### Differences in handgrip strength according to the age, sex and physical activity level

The HGS was compared across the four PA guideline adherence categories (i.e. “neither,” “aerobic only,” “resistance only,” and “combined”). The PA performance-based HGS was significantly different in all age groups and both sexes, except the 40 − 49 years old males (Table 2). Trends of the lowest and highest HGS in the “neither” and “combined” groups, respectively, were observed.

**Table 2:** HGS by sex, age group, and physical activity guideline adherence

Sex, age groups		HGS by physical activity guideline adherence category				
*Male (n* = *5646)*		Neither (*n* = 2501)	Aerobic only (*n* = 1395)	Resistance only (*n* = 609)	Combined (*n* = 801)	*p* value
Age group	40 − 49 years (*n* = 1368)	42.8 ± 7.2	43.0 ± 6.9	42.9 ± 6.4	43.9 ± 6.8	0.23
	50 − 59 years (*n* = 1378)	40.4 ± 6.3	41.0 ± 6.5	42.0 ± 6.8	41.8 ± 5.8	0.003
	60 − 69 years (*n* = 1323)	36.7 ± 6.5	37.0 ± 6.4	37.6 ± 5.9	39.4 ± 5.5	< 0.001
	70 years and above (*n* = 1237)	29.7 ± 6.9	31.8 ± 6.9	34.0 ± 6.2	33.9 ± 6.2	< 0.001
*Female (n* = *7168)*		Neither (*n* = 3920)	Aerobic only (*n* = 2002)	Resistance only (*n* = 404)	Combined (*n* = 529)	*p* value
Age group	40 − 49 years (*n* = 1774)	24.9 ± 4.5	25.7 ± 4.8	25.4 ± 4.7	26.3 ± 4.5	< 0.001
	50 − 59 years (*n* = 1852)	23.8 ± 4.3	24.3 ± 4.2	24.9 ± 4.1	25.3 ± 4.1	< 0.001
	60 − 69 years (*n* = 1642)	22.1 ± 4.5	23.0 ± 4.3	23.3 ± 4.0	23.8 ± 3.7	< 0.001
	70 years and above (*n* = 1587)	18.1 ± 4.5	19.2 ± 4.5	20.2 ± 4.3	21.0 ± 3.4	< 0.001

Physical activity guideline adherence categories are composed of four groups: “neither” (Moderate to vigorous physical activity, MVPA 0–149 min/week and resistance exercise 0–1 sessions/week); “aerobic only” (MVPA ≥ 150 min/week and resistance exercise 0–1 sessions/week); “resistance only” (resistance exercise ≥ 2 sessions/week and MVPA 0–149 min/week); and “combined” (MVPA ≥ 150 min/week and resistance exercise ≥ 2 sessions/week). *p* values were derived using analysis of variance

*HGS* handgrip strength

Additionally, to compare the HGS of subjects who never exercise and that of subjects who did exercise, but did not meet either exercise guidelines, we further divided “neither” group as “None” and “Some activity” group, respectively. HGS was significantly different between “None” and “Some activity” groups only for 70 years and above old females (Supplemental Table 2).

### Prevalence ratios for preserved handgrip strength according to physical activity guideline adherence categories

**Fig. 1:**
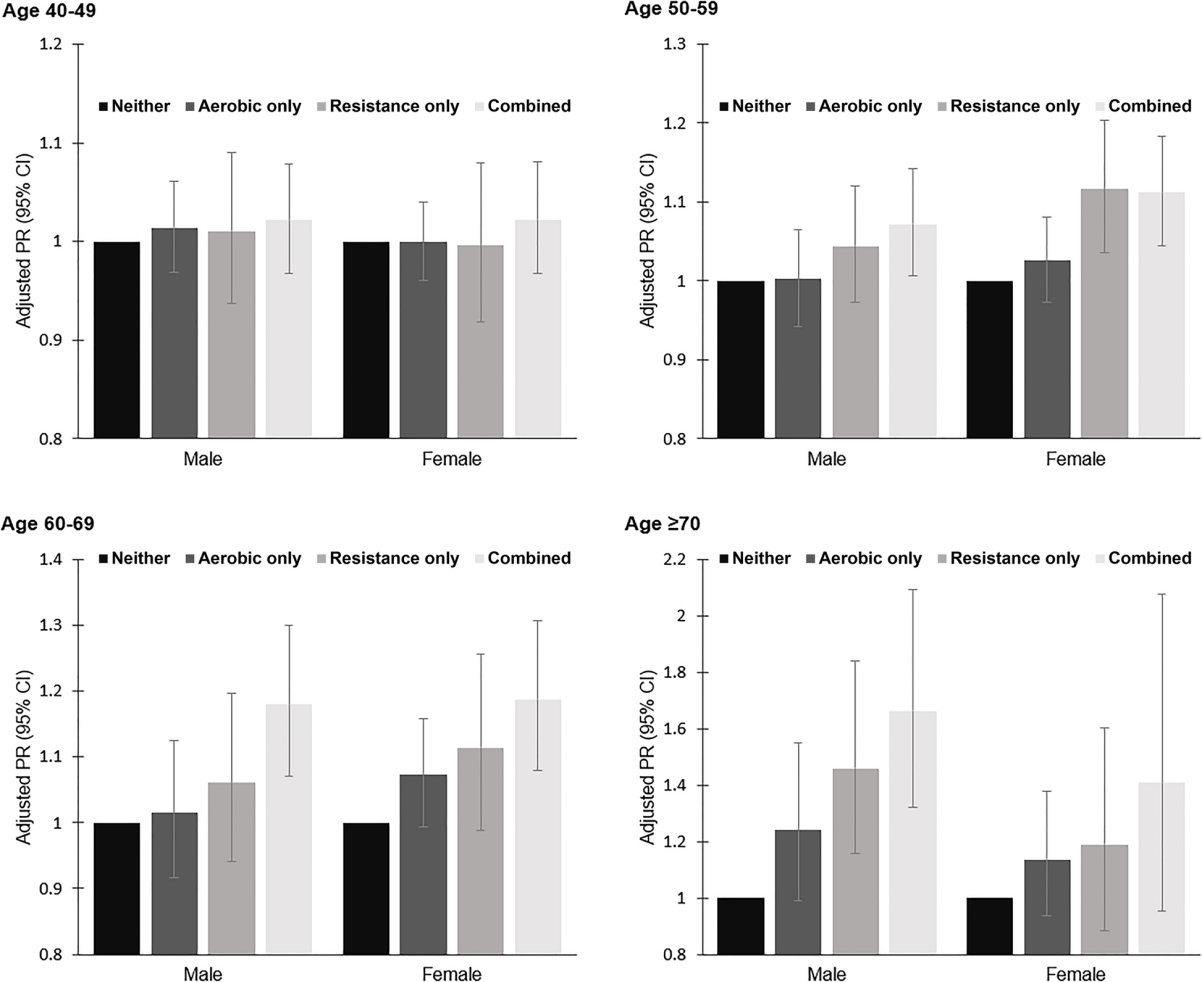
Adjusted PR (95% CI) for preserved HGS, according to physical activity guideline adherence categories. PR, prevalence ratio; HGS, handgrip strength

The unadjusted and adjusted prevalence ratios (APR) for preserved HGS across the PA guideline adherence categories, with “neither” group as a reference, are shown in Table 3 and Fig. 1. For male subjects in the 50 − 59 years age group and both sexes in the 60 − 69 years age group, which represents the middle-aged group, the APR was highest among those performing the “combined” exercise (male, age 50 − 59 years, APR = 1.072; male, age 60 − 69 years, APR = 1.180; female, age 60 − 69 years, APR = 1.188). For female subjects in the 50 − 59 years age group, higher APR was associated with both “resistance only” (APR = 1.116) and “combined” exercise groups (APR = 1.112). However, the youngest group (40 − 49 years) studied did not show significant differences in HGS according to the PA categories. For male subjects aged 70 years and over, the APR was highest among “combined” exercise group (APR = 1.664), followed by those in “resistance only” group (APR = 1.459). Conversely, for female subjects aged 70 years and over, no statistically significant differences across all the PA categories were observed.

**Table 3:** Unadjusted and adjusted prevalence ratios for preserved HGS across the physical activity guideline adherence categories

Characteristics	Model 1 (unadjusted)		Model 2 (adjusted)	
	^a^ **Unadjusted prevalence ratio (95% CI)**	***p*** ** value**	^b^ **Adjusted prevalence ratio (95% CI)**	***p*** ** value**
***Male***				
Age 40 − 49 years				
Neither	1		1	
Aerobic only	1.011 (0.968 − 1.054)	0.632	1.014 (0.969 − 1.060)	0.555
Resistance only	1.031 (0.967 − 1.100)	0.355	1.011 (0.937 − 1.090)	0.784
Combined	1.040 (0.991 − 1.090)	0.111	1.021 (0.967 − 1.078)	0.446
Age 50 − 59 years				
Neither	1		1	
Aerobic only	1.025 (0.972 − 1.081)	0.356	1.002 (0.943 − 1.065)	0.945
Resistance only	1.055 (0.990 − 1.124)	0.098	1.044 (0.973 − 1.120)	0.231
Combined	1.084 (1.027 − 1.143)	0.003	1.072 (1.006 − 1.142)	0.031
Age 60 − 69 years				
Neither	1		1	
Aerobic only	1.001 (0.910 − 1.101)	0.984	1.015 (0.917 − 1.124)	0.771
Resistance only	1.074 (0.961 − 1.201)	0.208	1.061 (0.941 − 1.197)	0.335
Combined	1.200 (1.100 − 1.310)	< 0.001	1.180 (1.070 − 1.300)	0.001
Age 70 years and above				
Neither	1		1	
Aerobic only	1.357 (1.098 − 1.678)	0.005	1.240 (0.991 − 1.551)	0.060
Resistance only	1.716 (1.385 − 2.126)	< 0.001	1.459 (1.158 − 1.839)	0.001
Combined	1.965 (1.597 − 2.417)	< 0.001	1.664 (1.323 − 2.092)	< 0.001
***Female***				
Age 40 − 49 years				
Neither	1		1	
Aerobic only	1.013 (0.976 − 1.052)	0.499	1.000 (0.961 − 1.040)	0.981
Resistance only	0.991 (0.913 − 1.074)	0.817	0.996 (0.919 − 1.080)	0.924
Combined	1.033 (0.980 − 1.089)	0.222	1.022 (0.967 − 1.080)	0.433
Age 50 − 59 years				
Neither	1		1	
Aerobic only	1.045 (1.000 − 1.092)	0.052	1.026 (0.973 − 1.081)	0.350
Resistance only	1.128 (1.063 − 1.198)	< 0.001	1.116 (1.036 − 1.203)	0.004
Combined	1.107 (1.048 − 1.171)	< 0.001	1.112 (1.045 − 1.184)	0.001
Age 60 − 69 years				
Neither	1		1	
Aerobic only	1.095 (1.027 − 1.168)	0.005	1.073 (0.994 − 1.159)	0.072
Resistance only	1.091 (0.978 − 1.218)	0.120	1.115 (0.989 − 1.256)	0.076
Combined	1.205 (1.107 − 1.313)	< 0.001	1.188 (1.079 − 1.307)	< 0.001
Age 70 years and above				
Neither	1		1	
Aerobic only	1.291 (1.118 − 1.491)	0.001	1.137 (0.938 − 1.377)	0.190
Resistance only	1.571 (1.265 − 1.952)	< 0.001	1.191 (0.884 − 1.603)	0.251
Combined	1.896 (1.471 − 2.443)	< 0.001	1.408 (0.953 − 2.079)	0.086

Physical activity guideline adherence categories are composed of four groups: “neither” (Moderate to vigorous physical activity, MVPA 0–149 min/week and resistance exercise 0–1 sessions/week); “aerobic only” (MVPA ≥ 150 min/week and resistance exercise 0–1 sessions/week); “resistance only” (resistance exercise ≥ 2 sessions/week and MVPA 0–149 min/week); and “combined” (MVPA ≥ 150 min/week and resistance exercise ≥ 2 sessions/week)

^a^Prevalence ratios were calculated using Poisson regression with a robust error variance

^b^Prevalence ratio adjusted for smoking, alcohol consumption, body mass index, protein intake, fat intake, carbohydrate intake, hypertension, dyslipidemia, and diabetes mellitus

*HGS* Handgrip strength, *CI* Confidence interval

## Discussion

The national representative data from middle-aged and older adults in Korea showed that only 14.2% in males and 7.4% in females of the studied population met the combined aerobic and resistance exercise guidelines recommended by WHO. Almost half (44.3% in males and 54.7% in females) of the population did not meet either exercise guidelines. People who engage in even slight amount of exercise showed significantly higher HGS compared to that of people with physical inactivity in a certain group of population (70 years and above old females). These results raise the need for the government’s action to promote PA for public health.

We investigated the relationship between PA patterns and HGS in middle-aged and older adults to give an insight for future studies regarding the most appropriate type of exercise for preserving HGS. Meeting both exercise guidelines in contrast to meeting aerobic only or resistance only was independently associated with preserved HGS in middle-aged adults. For adults aged 70 years and above, meeting either aerobic or resistance exercise guidelines showed a positive association with HGS; however, the prevalence ratio of preserved HGS was highest among participants meeting both exercise guidelines. Similar results were obtained in females (70 years and above), but not found significant after adjusting for other health-related variables. These findings suggest the additive benefits of combined aerobic and resistance exercise training for improving HGS and the need for optimized exercise prescription for individuals based on their age and sex. However, this hypothesis must be further tested in well-designed clinical trials.

In our study, adherence to both resistance and aerobic exercise guidelines were significantly associated with preserved HGS. Resistance exercise has been proven to be the most effective exercise type in terms of preventing and treating the sarcopenic process [13, 17, 19, 27, 34]. Although aerobic exercise is generally aimed at enhancing cardiovascular function with increased peak oxygen consumption, studies have found that both aerobic and resistance exercises contribute to alleviating the age-related decline of muscle strength and mass [21]. Previous studies have observed enhanced myofibrillar protein synthesis and muscle capillarization coupled with improved muscle strength and hypertrophy in response to aerobic exercise training (e.g., walking, running, cycling) [23, 35, 36]. Aerobic exercise induces mitochondrial adaptation and elicits changes in growth factor levels in skeletal muscle tissue toward positive protein turnover balance by reducing myostatin and increasing insulin-like growth factor-1 (IGF-1). Inversely, a lack of aerobic exercise increases lipid accumulation inside muscle fibers, and causes muscle cell death by increasing inflammatory cytokines and tumor necrosis factor-alpha (TNFα) [37–39].

Aerobic exercise with sufficient intensity and duration appears to be an adequate stimulus for muscle growth and attenuation of age-related reductions in muscle strength [40, 41]. Cross-sectional studies based on self-reported survey and accelerometry-derived physical activity data showed that an increase in MVPA replacing sedentary behavior and low-intensity PA was positively associated with HGS [42, 43]. According to a recent study also based on KNHANES (2014–2017) [44], adults performing less than 600 metabolic equivalents of aerobic exercise were associated with low HGS. Several clinical trials were conducted on middle-aged to older adults with comorbidities to investigate the positive effect of aerobic exercise on HGS [45–47]. Lo et al. [45] designed 12-week individualized aerobic exercise training combined with telephone-based motivational interviewing for middle-aged and older adults with multimorbidity, and demonstrated increased HGS in the intervention group compared to comparison or control group receiving only telephone-based motivational interviewing or usual care. Dos Anjos et al. [46] found that diabetic elderly women who performed regular aerobic training composed of walking and free calisthenic exercises achieved better functional capacity and increased HGS over the period of 10-week compared to baseline.

Consistent with the literature, our study showed that meeting aerobic exercise guideline was positively associated with preserved HGS, but it was only observed from older age groups (over 70 and 60 years in men and women, respectively) and before adjusting for other health-related variables (e.g., BMI and comorbidities). This result implies that the beneficial effects of aerobic exercise might be more prominent in the older population with underline health problems. Seong et al. [44] also showed similar findings, in which the odds ratio of lack of aerobic exercise for low HGS became higher among hypertension and diabetes patients than the odds ratio calculated from the total sample. A recent longitudinal study has proven multimorbidity itself is an important risk factor for sarcopenia [48]. As people with multimorbidity and physical inactivity consist of the most susceptible population to develop sarcopenia, the significance of metabolic and cardiovascular benefits from aerobic exercise [49–51], which in turn brings improved HGS, might be even greater in these population.

In this study, participants following the combined exercise guidelines showed the highest prevalence of preserved HGS. Contrary to our results, a combination of resistance and aerobic exercise has historically been associated with an “interference” effect, which indicates that physiologic adaptations elicited by aerobic and resistance exercise can interfere with each other when performed together. This means that improvements in muscle size and strength from resistance exercise can be attenuated by concurrently performed aerobic exercise [52, 53]. However, in the elderly population, the physiological effects attained from a combined exercise training were similar as compared to resistance or aerobic alone, indicating no evidence of the “interference” effect [24–26, 54]. Furthermore, a few previous studies indicated that combining two exercise modes, compared to either mode alone, was the most effective way to simultaneously improve muscle strength, aerobic fitness, and physical function in the elderly. Timmons et al. [24]. implemented 12-week time-matched intervention programs consisting of aerobic only, resistance only, and combined exercise in older adults, and found that the combined exercise program showed improved gait speed and lower limb strength than a single exercise mode. Additionally, Wood et al. [25]. found that the 12-week combined exercise regime enhanced arm strength and agility more than by the resistance or aerobic exercise alone.

There is ambiguity in establishing definitive and more specific exercise recommendations for sarcopenia [13]. This is because considerable heterogeneity exists in exercise intervention programs among randomized controlled trials conducted to assess the effects of exercise on sarcopenia parameters [14, 27], and studies on the comparative effectiveness of different exercise interventions are lacking [20]. However, recent evidences from systematic reviews and meta-analyses indicated that multi-component exercises incorporating aerobic, balance, gait, or flexibility trainings in addition to resistance training is the most effective way to alleviate functional decline and improve strength in frail older adults [16–18], which is consistent with our findings.

We also investigated the relationship between PA patterns and HGS according to sex and age groups. Younger age groups (age 40–49 years) did not show an association between exercise patterns and HGS; in the oldest age group (above 70 years), the effect of exercise on HGS was only observed in men. There has been a controversy regarding whether the age-related decline in muscle strength and response to exercises could be sex-specific [28, 55]. In most studies, males achieved greater absolute increases in muscle mass and strength in response to exercise training than females. Yet, others reported similar or higher relative strength gains in females. Previous studies have indicated that hormonal factors play a significant role in sex-based differences in neuromuscular adaptations to exercise, which include disparities in muscle fatigability, perfusion, and the time course of recovery [55]. Furthermore, previous studies investigated the relationship between PA and muscle volume and physical performance tests over the lifespan, and reported greater benefit of physical activity engagement in men than in women [56, 57]. This is in agreement with our results of stronger association of PA in preserving HGS in men than in women observed in the oldest age group. Further research is required to clarify the age- and sex-related differences in the development of sarcopenia, which would help in designing the most suitable exercise intervention programs individually according to these factors.

Our study has several limitations. First, we used self-reported measures of MVPA and resistance exercise performance, which is subject to recall bias. Under- or over-reporting due to social desirability, is possible. However, this limitation might not significantly bias our results because the KNHANES uses standardized self-report instruments for public health surveillance, and the recruited subject numbers were sufficient to decrease bias. Future studies with objective PA assessment using accelerometry can improve the accuracy of the collected data. However, objective recording of resistance exercise performance is still difficult with accelerometry. Second, the inferences of causality could not be determined because of the cross-sectional study design. Considering the cross-sectional design of our study, reverse causality or protopathic bias should be considered in interpreting our results. The healthy cohort effect might be suggested as in younger participants, the overall impact of comorbidities and physical activity behaviors over HGS can be lower than in older adults. Moreover, healthy older adults with higher HGS tend to engage in regular and more intensive PA because they have better physical and organic reserves to perform those exercises. Future longitudinal studies could better determine the temporal order of the association between the PA patterns and HGS. Third, information about muscle mass and physical performance (e.g., gait speed, timed up and go), which are also important components of sarcopenia diagnosis, was not available as data were derived from a standardized national survey that did not include those assessments. Further studies with a comprehensive assessment of all three components of sarcopenia in a large population are needed to better describe the relationship between the PA patterns and prevention of sarcopenia.

## Conclusion

Our study investigated the association between daily PA patterns and HGS using national representative data in Korea. Although there were some differences depending on age and sex, participants who reported habitually acquiring a sufficient amount of both aerobic and resistance exercises were associated with the highest prevalence of preserved HGS. Combined aerobic and resistance exercise training should be considered when conducting future clinical trials to design an effective preventive strategy against sarcopenia in middle-aged and older adults.

## Supplementary Information

**Additional file 1:Supplemental Table 1.** Unadjusted and adjusted prevalence ratios for preserved HGS across the physical activity guideline adherence categories (preserved HGS was defined by equal or above 27.0 kg for men and 16.0 kg for women based on the EWGSOP2). **Supplemental Table 2.** HGS by sex, age group, and physical activity guideline adherence.

## Data Availability

The dataset used in this study are available in a public, open access KNHANES repository. (https://knhanes.kdca.go.kr/knhanes/sub03/sub03_02_05.do).

## Abbreviations

PA: Physical activity
HGS: Handgrip strength
MVPA: Moderate-to-vigorous-intensity physical activity
PR: Prevalence ratio
APR: Adjusted prevalence ratio
