# Supplementary material for: The association of aerobic, resistance, and combined exercises with the handgrip strength of middle-aged and elderly Korean adults: a nationwide cross-sectional study
Source: BMC Geriatr. 2022 Aug 16;22:676. doi: 10.1186/s12877-022-03293-z (PMC9380318; doi:10.1186/s12877-022-03293-z)
Supplement: Supplementary file 1 — Additional file 1:Supplemental Table 1. Unadjusted and adjusted prevalence ratios for preserved HGS across the physical activity guideline adherence categories (preserved HGS was defined by equal or above 27.0 kg for men and 16.0 kg for women based on the EWGSOP2). Supplemental Table 2. HGS by sex, age group, and physical activity guideline adherence. [file 12877_2022_3293_MOESM1_ESM.docx]

**Supplemental Table 1.** Unadjusted and adjusted prevalence ratios for preserved HGS across the physical activity guideline adherence categories (preserved HGS was defined by equal or above 27.0 kg for men and 16.0 kg for women based on the EWGSOP2)

| **Characteristics** | | **Model 1 (unadjusted)** | | **Model 2 (adjusted)** | |
| --- | --- | --- | --- | --- | --- |
|  | | ***Unadjusted prevalence ratio (95% CI)** | ***p* value** | **†Adjusted prevalence ratio (95% CI)** | ***p* value** |
| ***Male*** | | | | | |
| Age 40−49 years | |  | | | |
|  | Neither | 1 |  | 1 |  |
|  | Aerobic only | 1.017 (1.005−1.029) | 0.007 | 1.007 (0.996−1.019) | 0.193 |
|  | Resistance only | 1.019 (1.008−1.030) | 0.001 | 1.011 (1.001−1.022) | 0.037 |
|  | Combined | 1.005 (0.986−1.024) | 0.614 | 0.999 (0.981−1.017) | 0.885 |
| Age 50−59 years | |  | | | |
|  | Neither | 1 |  | 1 |  |
|  | Aerobic only | 1.005 (0.986−1.024) | 0.627 | 0.993 (0.970−1.016) | 0.537 |
|  | Resistance only | 1.012 (0.992−1.033) | 0.244 | 1.006 (0.983−1.030) | 0.605 |
|  | Combined | 1.024 (1.012−1.037) | <0.001 | 1.016 (1.004−1.028) | 0.010 |
| Age 60−69 years | |  | | | |
|  | Neither | 1 |  | 1 |  |
|  | Aerobic only | 1.020 (0.987−1.055) | 0.238 | 1.018 (0.981−1.056) | 0.348 |
|  | Resistance only | 1.037 (1.000−1.075) | 0.052 | 1.049 (1.014−1.085) | 0.006 |
|  | Combined | 1.061 (1.033−1.090) | <0.001 | 1.046 (1.014−1.079) | 0.004 |
| Age 70 years and above | |  | | | |
|  | Neither | 1 |  | 1 |  |
|  | Aerobic only | 1.161 (1.066−1.266) | 0.001 | 1.117 (1.018−1.226) | 0.019 |
|  | Resistance only | 1.289 (1.187−1.400) | <0.001 | 1.224 (1.124−1.332) | <0.001 |
|  | Combined | 1.275 (1.167−1.392) | <0.001 | 1.210 (1.097−1.334) | <0.001 |
| ***Female*** | | | | | |
| Age 40−49 years | |  | | | |
|  | Neither | 1 |  | 1 |  |
|  | Aerobic only | 0.998 (0.982−1.014) | 0.771 | 0.994 (0.979−1.009) | 0.448 |
|  | Resistance only | 1.011 (0.989−1.034) | 0.323 | 1.003 (0.979−1.029) | 0.791 |
|  | Combined | 1.006 (0.985−1.026) | 0.599 | 1.005 (0.987−1.023) | 0.593 |
| Age 50−59 years | |  | | | |
|  | Neither | 1 |  | 1 |  |
|  | Aerobic only | 1.007 (0.990−1.025) | 0.397 | 1.002 (0.983−1.021) | 0.850 |
|  | Resistance only | 1.015 (0.989−1.043) | 0.257 | 1.007 (0.973−1.043) | 0.680 |
|  | Combined | 1.005 (0.978−1.032) | 0.746 | 1.001 (0.971−1.031) | 0.971 |
| Age 60−69 years | |  | | | |
|  | Neither | 1 |  | 1 |  |
|  | Aerobic only | 1.046 (1.016−1.077) | 0.003 | 1.038 (1.003−1.074) | 0.036 |
|  | Resistance only | 1.083 (1.049−1.119) | <0.001 | 1.068 (1.030−1.107) | <0.001 |
|  | Combined | 1.094 (1.066−1.123) | <0.001 | 1.076 (1.050−1.103) | <0.001 |
| Age 70 years and above | |  | | | |
|  | Neither | 1 |  | 1 |  |
|  | Aerobic only | 1.117 (1.040−1.200) | 0.002 | 1.125 (1.026−1.234) | 0.012 |
|  | Resistance only | 1.201 (1.076−1.339) | 0.001 | 1.149 (1.004−1.315) | 0.043 |
|  | Combined | 1.273 (1.115−1.453) | <0.001 | 1.214 (1.021−1.443) | 0.028 |

Physical activity guideline adherence categories are composed of four groups: “neither” (Moderate to vigorous physical activity, MVPA 0–149 min/week and resistance exercise 0–1 sessions/week); “aerobic only” (MVPA ≥150 min/week and resistance exercise 0–1 sessions/week); “resistance only” (resistance exercise ≥2 sessions/week and MVPA 0–149 min/week); and “combined” (MVPA ≥150 min/week and resistance exercise ≥2 sessions/week).

*Prevalence ratios were calculated using Poisson regression with a robust error variance.

†Prevalence ratio adjusted for smoking, alcohol consumption, body mass index, protein intake, fat intake, carbohydrate intake, hypertension, dyslipidemia, and diabetes mellitus.

*HGS* handgrip strength, *CI* confidence interval.

**Supplemental Table 2.** HGS by sex, age group, and physical activity guideline adherence

| **Sex, age groups** | | **HGS by physical activity guideline adherence category** | | | | | | |
| --- | --- | --- | --- | --- | --- | --- | --- | --- |
| *Male* *(n=5646)* | | None  (n=1592) | Some  Activity  (n=909) | Aerobic only  (n=1395) | Resistance only  (n=609) | Combined  (n=801) | *p* value | Post hoc* |
| Age group | 40−49 years | 43.2±7.4 | 42.2±6.8 | 43.0±6.9 | 42.9±6.4 | 43.9±6.8 | 0.115 |  |
|  | 50−59 years | 40.3±6.2^a^ | 40.4±6.6^b^ | 41.0±6.5^c^ | 42.0±6.8^d^ | 41.8±5.8^e^ | 0.008 | a-e |
|  | 60−69 years | 36.6±6.6^a^ | 36.8±6.1^b^ | 37.0±6.4^c^ | 37.6±5.9^d^ | 39.4±5.5^e^ | <0.001 | a-e, b-e, c-e |
|  | 70 years and above | 29.4±7.0^a^ | 30.2±6.8^b^ | 31.8±6.9^c^ | 34.0±6.2^d^ | 33.9±6.2^e^ | <0.001 | a-c, a-d, a-e, b-d, b-e, c-d, c-e |
| *Female* *(n=7168)* | | None  (n=2303) | Some activity  (n=1617) | Aerobic only  (n=2002) | Resistance only  (n=404) | Combined  (n=529) | *p* value | Post hoc |
| Age group | 40−49 years | 25.1±4.5^a^ | 24.7±4.6^b^ | 25.7±4.8^c^ | 25.4±4.7^d^ | 26.3±4.5^e^ | 0.001 | a-e, b-c, b-e |
|  | 50−59 years | 23.7±4.2^a^ | 23.9±4.5^b^ | 24.3±4.2^c^ | 24.9±4.1^d^ | 25.3±4.1^e^ | <0.001 | a-e, b-e |
|  | 60−69 years | 22.0±4.5^a^ | 22.1±4.4^b^ | 23.0±4.3^c^ | 23.3±4.0^d^ | 23.8±3.7^e^ | <0.001 | a-c, a-e, b-c, b-e |
|  | 70 years and above | 18.0±4.7^a^ | 18.8±4.2^b^ | 19.2±4.5^c^ | 20.2±4.3^d^ | 21.0±3.4^e^ | <0.001 | a-b, a-c, a-d, a-e |

*In post hoc analysis, a pair of two alphabets represents there is significant difference between those two groups.
